# Symptom trajectory types during recovery from acute exacerbation of COPD and their association with systemic inflammatory burden and prognosis

**DOI:** 10.3389/fcell.2026.1845159

**Published:** 2026-07-23

**Authors:** Xiang Zhou, Changqing Ye

**Affiliations:** 1 Department of Internal Medicine, Xianju Hospital of Traditional Chinese Medicine, Taizhou City, Zhejiang Province, China; 2 Department of Respiratory Medicine, Pujiang Hospital of Traditional Chinese Medicine, Pujiang, Zhejiang Province, China

**Keywords:** acute exacerbation, chronic obstructive pulmonary disease, C-reactive protein, group-based trajectory modeling, procalcitonin, prognosis, symptom trajectory, systemic inflammatory burden

## Abstract

**Objective:**

To identify symptom trajectory types during recovery from AECOPD using GBTM, explore the role of admission systemic inflammatory burden (assessed by CRP and PCT) in shaping recovery heterogeneity, and analyze the association between trajectory types and prognosis.

**Methods:**

A total of 216 AECOPD patients hospitalized between January 2023 and December 2024 were enrolled. CAT and mMRC were used to assess symptom changes at 7 time points from admission to 12 weeks post-discharge. Admission systemic inflammatory biomarkers (CRP and PCT) were collected as biomarkers of inflammatory burden. Sepsis was defined according to Sepsis-3 criteria as life-threatening organ dysfunction caused by a dysregulated host response to infection, identified by an acute SOFA score increase of ≥2 points. Available SOFA components included the respiratory sub-score (derived from PaO2/FiO2) and the coagulation sub-score; hepatic, renal, cardiovascular, and neurological sub-scores were not systematically collected. Accordingly, formal complete SOFA-based Sepsis-3 classification was not feasible; CRP and PCT are used herein as surrogate biomarkers of systemic inflammatory burden alongside partial SOFA data. GBTM was applied using the traj procedure in SAS 9.4 to identify symptom recovery trajectory types, and baseline characteristics and prognostic differences were compared among trajectory groups. Cox proportional hazards regression and Fine-Gray competing risk models were used to analyze the association between trajectory types and first re-exacerbation risk. Negative binomial regression was used to analyze the association between trajectory types and re-exacerbation and rehospitalization frequency. Multivariable logistic regression was used to identify independent predictors of non-rapid recovery trajectories.

**Results:**

Four symptom recovery trajectory types were identified: rapid recovery group (82 cases, 38.0%), slow recovery group (62 cases, 28.7%), persistent symptoms group (48 cases, 22.2%), and symptom rebound group (24 cases, 11.1%). Patients in non-rapid recovery groups exhibited significantly higher admission inflammatory biomarker levels (CRP and PCT) compared to the rapid recovery group (all P < 0.05). The median time to first re-exacerbation was 248, 156, 78, and 102 days, respectively (P < 0.001). Multivariable Cox regression showed that, using the rapid recovery group as reference, the slow recovery group (aHR = 1.62, 95%CI: 1.08–2.42), persistent symptoms group (aHR = 2.58, 95%CI: 1.68–3.96), and symptom rebound group (aHR = 2.24, 95%CI: 1.32–3.80) all had elevated first re-exacerbation risk (all P < 0.05). Negative binomial regression showed that non-rapid recovery groups had increased re-exacerbation and rehospitalization counts within 12 months (all P < 0.05). Higher admission CAT score (OR = 1.18), lower FEV1%pred (OR = 0.96), ≥2 exacerbations in the previous year (OR = 2.42), higher admission CRP (OR = 1.01, an admission inflammatory biomarker), and current smoking (OR = 2.15) were independent predictors of non-rapid recovery trajectory (all P < 0.05).

**Conclusion:**

Symptom trajectories during AECOPD recovery are heterogeneous, and elevated admission systemic inflammatory burden is associated with non-rapid recovery. Non-rapid recovery trajectories are associated with increased re-exacerbation risk and worse quality of life. Trajectory-based prognostic stratification combined with early inflammatory burden assessment may facilitate identification of high-risk patients and development of individualized follow-up management strategies.

## 1 Introduction

Chronic Obstructive Pulmonary Disease (COPD) is a common respiratory disease characterized by persistent airflow limitation, with acute exacerbation being a critical event in its disease course (Agustí et al., 2023). Acute exacerbations not only accelerate lung function decline but also significantly increase the risk of rehospitalization and mortality (Mathioudakis et al., 2022). Previous studies have primarily focused on triggers and treatment strategies for acute exacerbations, while understanding of the dynamic patterns of symptom changes during recovery remains insufficient (Hurst et al., 2022). Clinical practice has revealed substantial individual variation in recovery trajectories following acute exacerbation, with some patients experiencing prolonged or recurrent symptoms (Cai et al., 2022). AECOPD is frequently precipitated by respiratory infections that can drive systemic inflammatory responses; in a subset of patients, this dysregulated host response fulfills Sepsis-3 criteria—defined as life-threatening organ dysfunction caused by a dysregulated host response to infection, identified by an acute SOFA score increase of ≥2 points (Singer et al., 2016). Sepsis-related systemic inflammatory activation, reflected by biomarkers such as C-reactive protein (CRP) and procalcitonin (PCT), may compromise airway repair mechanisms and impede symptom resolution. However, as complete SOFA data (including hepatic, renal, cardiovascular, and neurological sub-scores) were not systematically collected in this study, formal Sepsis-3 classification was not feasible; CRP and PCT are used herein as surrogate biomarkers of systemic inflammatory burden alongside the available respiratory SOFA sub-score. Identifying different symptom recovery patterns and their prognostic significance is of great value for developing individualized follow-up protocols (Jo et al., 2020). This study employed Group-Based Trajectory Modeling (GBTM) to investigate symptom trajectory types during Acute Exacerbation of Chronic Obstructive Pulmonary Disease (AECOPD) recovery and their association with prognosis, with particular attention to the role of systemic inflammatory burden in shaping recovery heterogeneity.

## 2 Materials and methods

### 2.1 Study population

This study enrolled AECOPD patients hospitalized in the Department of Respiratory Medicine at our hospital between January 2023 and December 2024. The study protocol was approved by the hospital ethics committee.

Inclusion criteria: (1) Meeting the 2023 Global Initiative for Chronic Obstructive Lung Disease (GOLD) diagnostic criteria, i.e., post-bronchodilator Forced Expiratory Volume in 1 s (FEV1)/Forced Vital Capacity (FVC) < 0.70; (2) Meeting AECOPD diagnostic criteria, i.e., acute worsening of respiratory symptoms (dyspnea, cough, and/or sputum production) beyond normal day-to-day variations requiring medication change; (3) Age ≥40 years; (4) Hospital stay ≥48 h to ensure adequate acute treatment and sufficient time for baseline symptom assessment; (5) Completion of at least 12 months of follow-up after discharge.

Exclusion criteria: (1) Comorbid bronchial asthma, bronchiectasis, active pulmonary tuberculosis, lung cancer, or other respiratory diseases; (2) Severe heart failure (New York Heart Association [NYHA] functional class IV), severe hepatic or renal insufficiency, malignancy, or other serious underlying diseases; (3) Requiring invasive mechanical ventilation at admission or death within 48 h of admission; (4) Missing ≥3 time points (≥42.9%) for COPD Assessment Test (CAT) or modified Medical Research Council dyspnea scale (mMRC) scores; (5) Cognitive impairment or psychiatric disorders precluding symptom assessment cooperation.

Based on the above inclusion and exclusion criteria, screening through the hospital electronic medical record system and follow-up database initially identified 312 hospitalized AECOPD patients meeting diagnostic criteria. After excluding 42 cases with comorbid respiratory diseases, 28 cases with severe underlying diseases, 8 cases requiring invasive ventilation at admission or death within 48 h, 12 cases with excessive missing key variables, and 6 cases unable to cooperate with assessment, 216 eligible AECOPD patients were ultimately enrolled in this study.

### 2.2 Data collection

#### 2.2.1 Baseline data

Baseline data were collected through the electronic medical record system, including: (1) Demographic characteristics: age, sex, Body Mass Index (BMI); (2) Smoking history: smoking status (never smoker, former smoker, current smoker), smoking index (pack-years); (3) COPD-related indicators: disease duration, GOLD classification, number of exacerbations in the previous year, history of long-term oxygen therapy, history of long-term home noninvasive ventilation; (4) Comorbidities: assessed using the Charlson Comorbidity Index (CCI); (5) Laboratory indicators: first blood routine within 24 h of admission (white blood cell count, neutrophil percentage, lymphocyte count, hemoglobin), C-Reactive Protein (CRP), Procalcitonin (PCT), arterial blood gas analysis (pH, PaCO2, PaO2, PaO2/FiO2). Sepsis was defined according to Sepsis-3 criteria (Singer et al., 2016) as life-threatening organ dysfunction caused by a dysregulated host response to infection, operationalized by an acute SOFA score increase of ≥2 points. Of the six SOFA sub-scores, the respiratory component (derived from PaO2/FiO2 ratio) was available in all patients; however, hepatic (bilirubin), renal (creatinine), cardiovascular (MAP/vasopressor use), coagulation (platelet count), and neurological (GCS) sub-scores were not systematically collected, precluding complete SOFA-based Sepsis-3 classification. CRP and PCT were therefore used as surrogate biomarkers of systemic inflammatory burden consistent with sepsis-associated host responses, alongside the available partial SOFA data. This represents a limitation that will be addressed in future prospective studies. Sputum culture and bacterial susceptibility data were available for 148 of 216 patients (68.5%); owing to incomplete yield, these data were not incorporated into multivariable models but are summarized descriptively; (6) Pulmonary function: lung function data measured during the stable period within 6 months before the current acute exacerbation, with stable period defined as ≥4 weeks from the last exacerbation without acute worsening of respiratory symptoms, measuring FEV1, FEV1 percent predicted (FEV1%pred), FEV1/FVC; (7) Treatment information: antibiotic use, systemic corticosteroid use, noninvasive ventilation application, length of hospital stay. Antibiotic and corticosteroid use were recorded as binary variables; variation in drug class, duration, and dose represents a limitation of the current data.

#### 2.2.2 Symptom assessment

The mMRC and CAT were used to assess patient symptoms. Assessment time points included: admission (T0), discharge (T1), 1 week post-discharge (T2), 2 weeks post-discharge (T3), 4 weeks post-discharge (T4), 8 weeks post-discharge (T5), and 12 weeks post-discharge (T6). The mMRC scale scores range from 0 to 4, with higher scores indicating greater dyspnea severity. The CAT scale contains 8 items with a total score of 0–40, with higher scores indicating greater COPD impact on patient life.

#### 2.2.3 Prognostic outcomes

Primary outcomes: (1) Time to first re-exacerbation: defined as the interval from discharge to the next episode requiring additional treatment (including antibiotics and/or systemic corticosteroids) or hospitalization due to worsening respiratory symptoms, with ≥14 days elapsed since index discharge. For patients in the rapid recovery, slow recovery, and symptom rebound groups, re-exacerbation required symptom worsening after return to the individual’s post-discharge stable symptom level. For patients in the persistent symptoms group—who did not return to pre-admission baseline—re-exacerbation was operationally defined as a clinician-documented acute deterioration in respiratory status from the patient’s post-discharge stable symptom level, requiring initiation or escalation of antibiotic and/or systemic corticosteroid therapy, with ≥14 days since index discharge. Re-exacerbation events requiring hospitalization were cross-checked against the electronic medical record to minimize recall bias from telephone follow-up; (2) Number of re-exacerbations within 12 months; (3) Number of AECOPD-related rehospitalizations within 12 months.

Secondary outcomes: (1) 12-month all-cause mortality; (2) Emergency department visits; (3) Quality of life: assessed using the St. George’s Respiratory Questionnaire (SGRQ), with retrospective collection of SGRQ data at baseline and 12-month follow-up.

Follow-up was conducted through a combination of outpatient visits, telephone follow-up, and electronic medical record system queries, with follow-up cutoff date of 30 December 2025, or patient death date.

### 2.3 Symptom recovery trajectory analysis

#### 2.3.1 Trajectory modeling

GBTM was used to identify symptom change trajectory types during AECOPD recovery. This method can identify patient subgroups with similar symptom change patterns. CAT score was used as the primary symptom indicator for trajectory analysis, with mMRC score as the validation indicator.

GBTM was implemented using the traj procedure in SAS 9.4 (Jones and Nagin, 2013), with a censored normal distribution specified for the CAT outcome. Model construction process: (1) Models containing 1–6 trajectory groups were fitted sequentially; (2) The optimal model was selected based on Bayesian Information Criterion (BIC), Average Posterior Probability (APP), proportion of each group sample size, and clinical interpretability; (3) The ΔBIC >10 threshold for meaningful model improvement was applied following the conventions established by Jones et al. (2001) and Nagin (2005); APP >0.7 indicates good classification accuracy, and each group’s sample proportion should be >5%; (4) A quadratic polynomial function was selected for all four trajectory groups based on likelihood ratio testing, as it provided adequate fit without overfitting.

#### 2.3.2 Trajectory group validation

The following methods were used to validate the robustness of trajectory grouping: (1) Independent GBTM analysis using mMRC scores, comparing consistency with CAT score-derived trajectory groupings, with Cohen’s Kappa coefficient assessing agreement between the two grouping schemes (Kappa >0.6 indicates good agreement); (2) Calculating entropy for each trajectory group to assess classification certainty (entropy >0.8 indicates good classification); (3) Comparing baseline characteristics and prognostic outcomes across trajectory groups to validate clinical significance of groupings.

#### 2.3.3 Trajectory type naming

Trajectory groups were named based on CAT score change characteristics, primarily considering: (1) Baseline symptom severity; (2) Recovery speed (rapid/slow); (3) Final symptom level; (4) Presence of persistent symptoms or rebound.

### 2.4 Statistical analysis

Statistical analysis was performed using R 4.3.0 and SPSS 26.0 software. Continuous variables with normal distribution were expressed as mean ± standard deviation (x̄±s), with intergroup comparisons using one-way analysis of variance (ANOVA) and post-hoc pairwise comparisons using Bonferroni correction; non-normally distributed variables were expressed as median (interquartile range) [M(P25, P75)], with intergroup comparisons using Kruskal–Wallis H test and post-hoc pairwise comparisons using Dunn-Bonferroni correction. Categorical variables were expressed as count (percentage) [n(%)], with intergroup comparisons using χ^2^ test or Fisher’s exact test, and post-hoc pairwise comparisons using χ^2^ partitioning with Bonferroni correction, with corrected significance level α = 0.0083.

Follow-up was completed for all 216 patients through outpatient visits, telephone contact, and electronic medical record queries, with no loss to follow-up during the 12-month observation period. GBTM was used to identify symptom recovery trajectory types, with BIC, APP (>0.7), proportion of each group sample size (>5%), and entropy (>0.8) determining the optimal number of trajectory groups. A sensitivity analysis comparing the 12 excluded patients (≥3 missing time points) against the included sample found no statistically significant differences in admission CAT score, FEV1%pred, CRP, or exacerbation history (all P > 0.05), suggesting that missing-data exclusion did not introduce systematic bias. Cohen’s Kappa coefficient was used to assess consistency between CAT and mMRC score trajectory groupings. Survival analysis used the Kaplan-Meier method for survival curves, with Log-rank test comparing intergroup differences. Cox proportional hazards regression was used to analyze the association between trajectory types and first re-exacerbation risk, with Schoenfeld residuals testing the proportional hazards assumption; the Fine-Gray model was used for competing risk analysis (with death as the competing event). Negative binomial regression was used to analyze the association between trajectory types and re-exacerbation and rehospitalization counts. Multivariable logistic regression was used to identify independent predictors of non-rapid recovery trajectories. Two-sided tests were used, with P < 0.05 considered statistically significant. No formal *a priori* power calculation was performed; this is acknowledged as a limitation, particularly for the symptom rebound subgroup (n = 24) where the event-per-variable ratio falls below the recommended threshold of 10 events per predictor, and results for this subgroup should be interpreted with caution.

## 3 Results

### 3.1 Identification of symptom recovery trajectory types

#### 3.1.1 Trajectory model selection

GBTM analysis based on CAT scores showed that as the number of trajectory groups increased, BIC values gradually decreased. When trajectory groups increased from 3 to 4, the BIC difference was 68.3 (>10), indicating improved model fit; when increasing from 4 to 5 groups, the BIC difference was 7.2 (<10), and one group in the 5-group model had a sample proportion of only 4.2% (<5%). Therefore, the 4-group trajectory model was ultimately selected (Table 1).

**TABLE 1:** Comparison of fit indices for models with different numbers of trajectory groups.

Number of trajectory groups	BIC	ΔBIC	Minimum group proportion (%)	Average posterior probability range	Entropy
1	−2847.6	—	100	1	^a^
2	−2692.3	155.3	36.1	0.89–0.94	0.82
3	−2598.5	93.8	18.5	0.84–0.91	0.84
4	−2530.2	68.3	11.1	0.81–0.93	0.86
5	−2523	7.2	4.2	0.72–0.91	0.79
6	−2518.4	4.6	3.7	0.68–0.89	0.76

BIC, bayesian information criterion; ΔBIC, BIC, difference from the previous model.

^a^ Entropy cannot be calculated for single-group models.

#### 3.1.2 Trajectory type characteristics

Based on the 4-group trajectory model, 216 AECOPD patients were classified into the following 4 symptom recovery trajectory types (Table 2).

1. Rapid recovery group (n = 82, 38.0%): Moderate-to-high CAT score at admission (24.8 ± 4.2), with rapid symptom improvement after treatment. CAT score decreased to 12.6 ± 3.8 at 4 weeks post-discharge and was lowest at 12 weeks (11.6 ± 3.5).
2. Slow recovery group (n = 62, 28.7%): Higher CAT score at admission (27.3 ± 4.6), with slower symptom improvement, gradually stabilizing by 8 weeks post-discharge (16.8 ± 4.1), and CAT score of 14.5 ± 3.9 at 12 weeks.
3. Persistent symptoms group (n = 48, 22.2%): Highest CAT score at admission (30.5 ± 4.8), with limited symptom improvement after treatment, remaining at elevated levels post-discharge, with CAT score still reaching 21.3 ± 4.6 at 12 weeks.
4. Symptom rebound group (n = 24, 11.1%): Higher CAT score at admission (28.1 ± 4.4), with initial symptom improvement within 2 weeks post-discharge (18.2 ± 3.6 at T3), followed by symptom re-worsening. CAT score rose to 24.6 ± 4.2 at 12 weeks, approximately 88% of admission levels.

**TABLE 2:** Comparison of CAT Scores at Different Time Points Among Trajectory Groups (points, x̄±s).

Time point	Rapid recovery group (n = 82)	Slow recovery group (n = 62)	Persistent symptoms group (n = 48)	Symptom rebound group (n = 24)	F Value	P Value
T0 (Admission)	24.8 ± 4.2	27.3 ± 4.6	30.5 ± 4.8	28.1 ± 4.4	15.72	<0.001
T1 (Discharge)	18.5 ± 3.6	22.6 ± 4.2	26.8 ± 4.5	22.3 ± 4.1	28.64	<0.001
T2 (1 week post-discharge)	15.2 ± 3.4	20.8 ± 4.0	25.2 ± 4.3	19.5 ± 3.8	42.38	<0.001
T3 (2 weeks post-discharge)	13.4 ± 3.2	19.2 ± 3.9	24.1 ± 4.2	18.2 ± 3.6	51.26	<0.001
T4 (4 weeks post-discharge)	12.6 ± 3.8	17.5 ± 3.8	23.4 ± 4.4	21.4 ± 3.8	48.93	<0.001
T5 (8 weeks post-discharge)	11.8 ± 3.5	16.8 ± 4.1	22.5 ± 4.5	23.2 ± 4.0	56.17	<0.001
T6 (12 weeks post-discharge)	11.6 ± 3.5	14.5 ± 3.9	21.3 ± 4.6	24.6 ± 4.2	68.42	<0.001

CAT, COPD, assessment test; T0–T6 represent admission, discharge, and 1, 2, 4, 8, and 12 weeks post-discharge, respectively.

#### 3.1.3 Trajectory group validation

Independent GBTM analysis based on mMRC scores also identified 4 trajectory types, with moderate-to-good consistency with CAT score-derived groupings (Cohen’s Kappa = 0.72, P < 0.001). The overall entropy for the 4-group model was 0.86, indicating good classification certainty. APP for each trajectory group were: rapid recovery group 0.93, slow recovery group 0.86, persistent symptoms group 0.84, and symptom rebound group 0.81, all >0.7 (Table 3). Supplementary P-values for pairwise comparisons of mMRC scores among trajectory groups are shown in Table 4.

**TABLE 3:** Comparison of mMRC Scores at Different Time Points Among Trajectory Groups (points, M[P25, P75]).

Time point	Rapid recovery group (n = 82)	Slow recovery group (n = 62)	Persistent symptoms group (n = 48)	Symptom rebound group (n = 24)	H Value	P Value	Post-hoc comparison
T0 (Admission)	3[2, 3]	3[3, 4]	4[3, 4]	3[3, 4]	28.64	<0.001	A < C
T1 (Discharge)	2[2, 3]	3[2, 3]	3[3, 4]	3[2, 3]	35.72	<0.001	A < C
T2 (1 week post-discharge)	2[1, 2]	2[2, 3]	3[3, 4]	2[2, 3]	52.18	<0.001	A < B,C
T3 (2 weeks post-discharge)	1[1, 2]	2[2, 3]	3[2, 3]	2[2, 3]	58.34	<0.001	A < B,C
T4 (4 weeks post-discharge)	1[1, 2]	2[2, 2]	3[2, 3]	2[2, 3]	62.47	<0.001	A < B,C
T5 (8 weeks post-discharge)	1[1, 2]	2[1, 2]	3[2, 3]	3[2, 3]	68.29	<0.001	A < B,C
T6 (12 weeks post-discharge)	1[1, 2]	2[1, 2]	3[2, 3]	3[3, 4]	71.84	<0.001	A < C,D

mMRC, modified Medical Research Council dyspnea scale; A = Rapid recovery group, B=Slow recovery group, C=Persistent symptoms group, D = Symptom rebound group; Post-hoc comparisons used Dunn-Bonferroni correction, with corrected P < 0.0083 considered statistically significant; only statistically significant pairwise comparisons after correction are listed.

**TABLE 4:** Supplementary P-values for Pairwise Comparisons of mMRC Scores Among Trajectory Groups (Dunn-Bonferroni Correction).

Time point	A vs. B	A vs. C	A vs. D	B Vs. C	B Vs. D	C Vs. D
T0	0.068	<0.001	0.052	0.056	0.485	0.326
T1	0.058	<0.001	0.056	0.052	0.524	0.186
T2	0.006	<0.001	0.012	0.012	0.368	0.124
T3	0.003	<0.001	0.01	0.01	0.286	0.098
T4	0.002	<0.001	0.006	0.002	0.242	0.086
T5	0.004	<0.001	0.003	0.01	0.068	0.156
T6	0.012	<0.001	<0.001	0.024	0.086	0.086

A = Rapid recovery group, B=Slow recovery group, C=Persistent symptoms group, D = Symptom rebound group; Corrected P < 0.0083 considered statistically significant.

### 3.2 Comparison of baseline characteristics among trajectory groups

#### 3.2.1 Demographic characteristics and smoking history

There were no statistically significant differences in age, sex, or BMI among the 4 groups (P > 0.05). Smoking status distribution differed significantly among groups (P = 0.023). Post-hoc pairwise comparisons using χ^2^ partitioning (Bonferroni-corrected α = 0.0083) showed that the proportion of current smokers in the persistent symptoms group was significantly higher than in the rapid recovery group (P = 0.006). Smoking index differed significantly among groups (P = 0.008), with post-hoc comparisons showing the persistent symptoms group had significantly higher smoking index than the rapid recovery group (P = 0.001) (Table 5).

**TABLE 5:** Comparison of baseline characteristics among trajectory groups.

Variable	Rapid recovery group (n = 82)	Slow recovery group (n = 62)	Persistent symptoms group (n = 48)	Symptom rebound group (n = 24)	Statistic	P Value	Post-hoc comparison
Demographic characteristics							
Age (years, x̄±s)	67.2 ± 8.4	68.5 ± 9.1	69.8 ± 8.7	68.1 ± 9.3	F = 0.89	0.448	—
Male [n(%)]	62(75.6)	48(77.4)	38(79.2)	18(75.0)	χ^2^ = 0.28	0.964	—
BMI (kg/m^2^, x̄±s)	22.8 ± 3.6	22.1 ± 3.8	21.5 ± 4.1	21.9 ± 3.9	F = 1.24	0.296	—
Smoking history							
Smoking status [n(%)]	​	​	​	​	χ^2^ = 14.72	0.023	A < C^a
Never smoker	15(18.3)	10(16.1)	5(10.4)	3(12.5)	​	​	​
Former smoker	48(58.5)	32(51.6)	22(45.8)	10(41.7)	​	​	​
Current smoker	19(23.2)	20(32.3)	21(43.8)	11(45.8)	​	​	​
Smoking index (pack-years, M[P25,P75])	35[20, 48]	40[25, 55]	48[32, 62]	42[26, 56]	H = 11.82	0.008	A < C
COPD-related indicators							
Disease duration (years, M[P25,P75])	8[5, 12]	9[6, 14]	10[6, 15]	9[5, 13]	H = 5.21	0.156	—
GOLD classification [n(%)]	​	​	​	​	χ^2^ = 32.68	<0.001	A < C; B < C^b
Grade I	8(9.8)	3(4.8)	1(2.1)	1(4.2)	​	​	​
Grade II	38(46.3)	22(35.5)	10(20.8)	6(25.0)	​	​	​
Grade III	28(34.1)	28(45.2)	24(50.0)	12(50.0)	​	​	​
Grade IV	8(9.8)	9(14.5)	13(27.1)	5(20.8)	​	​	​
Exacerbations in previous year (times, M[P25,P75])	1[0, 2]	2[1, 3]	2[1, 4]	2[1, 3]	H = 24.56	<0.001	A < B,C,D
History of long-term oxygen therapy [n(%)]	12(14.6)	16(25.8)	20(41.7)	8(33.3)	χ^2^ = 14.86	0.002	A < C
History of long-term home NIV [n(%)]	5(6.1)	8(12.9)	12(25.0)	4(16.7)	χ^2^ = 10.12	0.018	—^c
Comorbidities							
CCI score (points, M[P25,P75])	2[1, 3]	2[1, 4]	3[2, 5]	3[2, 4]	H = 13.28	0.004	A < C
Laboratory indicators	​	​	​	​	​	​	​
WBC count (×10^9^/L, x̄±s)	9.8 ± 3.2	10.6 ± 3.5	11.8 ± 4.1	11.2 ± 3.8	F = 3.68	0.012	A < C
Neutrophil percentage (%, x̄±s)	72.4 ± 10.2	75.8 ± 9.6	78.6 ± 8.9	76.9 ± 9.4	F = 4.21	0.006	A < C
Lymphocyte count (×10^9^/L, x̄±s)	1.32 ± 0.48	1.25 ± 0.52	1.18 ± 0.56	1.22 ± 0.51	F = 0.82	0.485	—
Hemoglobin (g/L, x̄±s)	132.5 ± 18.4	128.6 ± 19.2	126.8 ± 20.5	129.4 ± 18.8	F = 0.94	0.421	—
CRP (mg/L, M[P25,P75])	28.5[12.4, 52.6]	42.3[18.6, 78.4]	68.4[32.5, 112.8]	54.6[24.8, 96.2]	H = 28.74	<0.001	A < C; B < C
PCT (ng/mL, M[P25,P75])	0.12[0.06, 0.28]	0.18[0.08, 0.42]	0.32[0.12, 0.68]	0.24[0.10, 0.52]	H = 14.06	0.003	A < C
pH (x̄±s)	7.38 ± 0.05	7.37 ± 0.06	7.35 ± 0.07	7.36 ± 0.06	F = 3.18	0.024	—^d
PaCO2 (mmHg, x̄±s)	48.2 ± 8.6	51.4 ± 10.2	56.8 ± 12.4	53.6 ± 11.2	F = 3.92	0.009	A < C
PaO2 (mmHg, x̄±s)	62.4 ± 10.5	60.8 ± 11.2	58.6 ± 12.8	59.4 ± 11.6	F = 1.12	0.342	—
PaO2/FiO2 (mmHg, x̄±s)	248.6 ± 52.4	238.4 ± 58.6	228.5 ± 64.2	234.2 ± 60.8	F = 1.36	0.256	—
Pulmonary function (stable period)							
FEV1 (L, x̄±s)	1.42 ± 0.48	1.18 ± 0.42	0.92 ± 0.38	1.05 ± 0.40	F = 13.86	<0.001	A > B,C,D
FEV1%pred (%, x̄±s)	52.6 ± 14.8	44.2 ± 13.6	36.8 ± 12.4	40.5 ± 13.2	F = 14.52	<0.001	A>C,D
FEV1/FVC (%, x̄±s)	52.4 ± 8.6	48.6 ± 9.2	44.2 ± 10.4	46.8 ± 9.8	F = 5.12	0.002	A>C
Treatment information							
Antibiotic use [n(%)]	76(92.7)	60(96.8)	47(97.9)	24(100.0)	—	0.312†	—
Systemic corticosteroid use [n(%)]	62(75.6)	50(80.6)	40(83.3)	20(83.3)	χ^2^ = 1.58	0.664	—
NIV application [n(%)]	14(17.1)	18(29.0)	26(54.2)	8(33.3)	χ^2^ = 18.64	<0.001	A < C; B < C
Length of hospital stay (days, M[P25,P75])	8[6, 10]	10[8, 13]	12[9, 16]	11[8, 14]	H = 32.86	<0.001	A < B,C; B < C

BMI, body mass index; GOLD, global initiative for chronic obstructive lung disease; NIV, noninvasive ventilation; CCI, charlson comorbidity index; WBC, white blood cell; CRP, C-reactive protein; PCT, procalcitonin; FEV1, forced expiratory volume in 1 s; FEV1%pred, FEV1 percent predicted; FVC, forced vital capacity; †Fisher’s exact test; A = Rapid recovery group, B = Slow recovery group, C = Persistent symptoms group, D = Symptom rebound group; Post-hoc comparisons: continuous variables used Bonferroni correction (normal distribution) or Dunn-Bonferroni correction (non-normal distribution); categorical variables used χ^2^ partitioning or Fisher’s exact test with Bonferroni correction; corrected P < 0.0083 considered statistically significant; only statistically significant pairwise comparisons after correction are listed; ^a Refers to comparison of current smoking proportions; ^b Refers to comparison of GOLD III–IV, proportions; ^c All pairwise comparisons did not reach statistical significance after correction; ^d Although overall comparison P < 0.05, all pairwise comparisons did not reach statistical significance after correction.

#### 3.2.2 COPD-related indicators

There was no statistically significant difference in COPD disease duration among the 4 groups (P = 0.156). GOLD classification distribution differed significantly among groups (P < 0.001). Post-hoc pairwise comparisons using χ^2^ partitioning (Bonferroni-corrected α = 0.0083) showed that the proportion of GOLD III–IV patients in the persistent symptoms group was significantly higher than in the rapid recovery group (P < 0.001) and slow recovery group (P = 0.005). The number of exacerbations in the previous year differed significantly among groups (P < 0.001), with post-hoc comparisons showing the rapid recovery group was significantly lower than the slow recovery group (P = 0.006), persistent symptoms group (P < 0.001), and symptom rebound group (P = 0.006). History of long-term oxygen therapy (P = 0.002) and history of long-term home noninvasive ventilation (P = 0.018) both differed significantly among groups. Post-hoc pairwise comparisons using Fisher’s exact test with Bonferroni correction showed that the proportion with history of long-term oxygen therapy in the persistent symptoms group was significantly higher than in the rapid recovery group (P = 0.001); pairwise comparisons for history of long-term home noninvasive ventilation did not reach statistical significance after correction (all P > 0.0083) (Tables 5, 7).

**TABLE 6:** Supplementary summary of P-values for pairwise comparisons of baseline characteristics.

Comparison Groups	Smoking Index	Exacerbations in Previous Year	CCI Score	WBC Count	Neutrophil %	CRP	PCT	pH	PaCO2	FEV1	FEV1%pred	FEV1/FVC	Length of Stay
A vs. B	0.124	0.006	0.156	0.086	0.068	0.052	0.086	0.124	0.068	0.008	0.012	0.056	0.006
A vs. C	0.001	<0.001	0.002	0.006	0.003	<0.001	0.001	0.018	0.005	<0.001	<0.001	0.002	<0.001
A vs. D	0.058	0.006	0.052	0.052	0.086	0.056	0.068	0.086	0.058	0.004	0.003	0.056	0.012
B vs. C	0.086	0.124	0.068	0.142	0.168	0.008	0.068	0.186	0.042	0.016	0.012	0.068	0.006
B vs. D	0.368	0.486	0.312	0.426	0.524	0.186	0.412	0.386	0.426	0.186	0.268	0.386	0.286
C vs. D	0.286	0.386	0.425	0.486	0.562	0.124	0.286	0.524	0.286	0.168	0.224	0.312	0.324

A = Rapid recovery group, B = Slow recovery group, C = Persistent symptoms group, D = Symptom rebound group; CCI, charlson comorbidity index; CRP, C-reactive protein; PCT, procalcitonin; FEV1, forced expiratory volume in 1 s; FEV1%pred, FEV1 percent predicted; FVC, forced vital capacity; Corrected P < 0.0083 considered statistically significant.

**TABLE 7:** Supplementary 2 summary of P-values for pairwise comparisons of categorical variables.

Comparison Groups	Smoking Status (Current vs. Non-current Smoker)	GOLD Classification (Grade III–IV vs. Grade I–II)	History of Long-term Oxygen Therapy	History of Long-term Home NIV	NIV Application
A vs. B	0.124	0.086	0.068	0.124	0.068
A vs. C	0.006	<0.001	0.001	0.012	<0.001
A vs. D	0.024	0.032	0.042	0.086	0.086
B vs. C	0.142	0.005	0.056	0.068	0.006
B vs. D	0.186	0.286	0.424	0.524	0.624
C vs. D	0.524	0.386	0.386	0.286	0.068

A = Rapid recovery group, B = Slow recovery group, C = Persistent symptoms group, D = Symptom rebound group; NIV, noninvasive ventilation; χ^2^ partitioning or Fisher’s exact test used, Bonferroni-corrected P < 0.0083 considered statistically significant.

#### 3.2.3 Comorbidities and laboratory indicators

CCI scores differed significantly among groups (P = 0.004), with post-hoc comparisons showing the persistent symptoms group had significantly higher CCI scores than the rapid recovery group (P = 0.002). For inflammatory markers at admission, white blood cell count (P = 0.012), neutrophil percentage (P = 0.006), CRP (P < 0.001), and PCT (P = 0.003) all differed significantly among groups. Post-hoc comparisons showed: the persistent symptoms group had significantly higher white blood cell count than the rapid recovery group (P = 0.006); the persistent symptoms group had significantly higher neutrophil percentage than the rapid recovery group (P = 0.003); the persistent symptoms group had significantly higher CRP than the rapid recovery group (P < 0.001) and slow recovery group (P = 0.008); the persistent symptoms group had significantly higher PCT than the rapid recovery group (P = 0.001). Lymphocyte count and hemoglobin did not differ significantly among groups (P > 0.05). For arterial blood gas analysis, pH (P = 0.024) and PaCO2 (P = 0.009) differed significantly among groups. Post-hoc comparisons showed the persistent symptoms group had significantly higher PaCO2 than the rapid recovery group (P = 0.005); pairwise comparisons for pH did not reach statistical significance after Bonferroni correction (all P > 0.0083); PaO2 and PaO2/FiO2 did not differ significantly among groups (P > 0.05) (Tables 5, 6).

#### 3.2.4 Pulmonary function and treatment information

For stable-period pulmonary function indicators, FEV1 (P < 0.001), FEV1%pred (P < 0.001), and FEV1/FVC (P = 0.002) all differed significantly among groups. Post-hoc comparisons showed: the rapid recovery group had significantly higher FEV1 than the slow recovery group (P = 0.008), persistent symptoms group (P < 0.001), and symptom rebound group (P = 0.004); the rapid recovery group had significantly higher FEV1%pred than the persistent symptoms group (P < 0.001) and symptom rebound group (P = 0.003); the rapid recovery group had significantly higher FEV1/FVC than the persistent symptoms group (P = 0.002). For treatment, antibiotic use rates did not differ significantly among groups (P = 0.312); systemic corticosteroid use rates did not differ significantly among groups (P = 0.664); noninvasive ventilation application rates differed significantly among groups (P < 0.001). Post-hoc pairwise comparisons using χ^2^ partitioning (Bonferroni-corrected α = 0.0083) showed that the noninvasive ventilation application rate in the persistent symptoms group was significantly higher than in the rapid recovery group (P < 0.001) and slow recovery group (P = 0.006); length of hospital stay differed significantly among groups (P < 0.001). Post-hoc comparisons showed: the persistent symptoms group had significantly longer hospital stay than the rapid recovery group (P < 0.001) and slow recovery group (P = 0.006); the slow recovery group had significantly longer hospital stay than the rapid recovery group (P = 0.006); the difference between the symptom rebound group and rapid recovery group did not reach statistical significance (P = 0.012) (Table 5).

### 3.3 Comparison of prognostic outcomes among trajectory groups

#### 3.3.1 Primary outcomes

During the 12-month follow-up period, 162 of 216 patients (75.0%) experienced re-exacerbation. Re-exacerbation rates by group were: rapid recovery group 63.4% (52/82), slow recovery group 82.3% (51/62), persistent symptoms group 81.3% (39/48), and symptom rebound group 83.3% (20/24), with statistically significant differences among groups (χ^2^ = 12.86, P = 0.005). Post-hoc pairwise comparisons using χ^2^ partitioning (Bonferroni-corrected α = 0.0083) showed that the slow recovery group (P = 0.006), persistent symptoms group (P = 0.008), and symptom rebound group (P = 0.006) all had significantly higher re-exacerbation rates than the rapid recovery group; differences between other group pairs were not statistically significant (all P > 0.0083). In the persistent symptoms group, 8 patients died during follow-up, of whom 5 experienced exacerbation before death and 3 died without prior exacerbation; in the symptom rebound group, 2 patients died during follow-up, of whom 1 experienced exacerbation before death and 1 died without prior exacerbation. The above re-exacerbation rates include patients who experienced exacerbation before death, with denominators being the total number of patients in each group.

Kaplan-Meier survival analysis showed statistically significant differences in time to first re-exacerbation among trajectory groups (Log-rank χ^2^ = 38.62, P < 0.001). Median time to first re-exacerbation was 248 days (95%CI: 218–278 days) for the rapid recovery group, 156 days (95%CI: 124–188 days) for the slow recovery group, 78 days (95%CI: 58–98 days) for the persistent symptoms group, and 102 days (95%CI: 72–132 days) for the symptom rebound group. Pairwise comparisons (Log-rank test, Bonferroni-corrected α = 0.0083) showed: the rapid recovery group had significantly longer median time to first re-exacerbation than the slow recovery group (P < 0.001), persistent symptoms group (P < 0.001), and symptom rebound group (P < 0.001); the slow recovery group was significantly longer than the persistent symptoms group (P = 0.002); differences between the slow recovery group and symptom rebound group (P = 0.048) and between the persistent symptoms group and symptom rebound group (P = 0.186) were not statistically significant.

For the number of re-exacerbations within 12 months, the rapid recovery group had a median of 1 (IQR: 0–2), slow recovery group 2 (IQR: 1–3), persistent symptoms group 3 (IQR: 2–4), and symptom rebound group 2 (IQR: 1–4), with statistically significant differences among groups (H = 48.62, P < 0.001). Post-hoc pairwise comparisons (Dunn-Bonferroni correction) showed: the rapid recovery group had significantly fewer re-exacerbations than the slow recovery group (P = 0.003), persistent symptoms group (P < 0.001), and symptom rebound group (P = 0.005); the slow recovery group had significantly fewer than the persistent symptoms group (P = 0.006); differences between the slow recovery group and symptom rebound group (P = 0.324) and between the persistent symptoms group and symptom rebound group (P = 0.142) were not statistically significant.

For the number of AECOPD-related rehospitalizations within 12 months, the rapid recovery group had a median of 0 (IQR: 0–1), slow recovery group 1 (IQR: 0–2), persistent symptoms group 2 (IQR: 1–3), and symptom rebound group 1 (IQR: 0–2), with statistically significant differences among groups (H = 38.94, P < 0.001). Post-hoc pairwise comparisons (Dunn-Bonferroni correction) showed: the rapid recovery group had significantly fewer rehospitalizations than the slow recovery group (P = 0.004), persistent symptoms group (P < 0.001), and symptom rebound group (P = 0.006); the slow recovery group had significantly fewer than the persistent symptoms group (P = 0.003); differences between the slow recovery group and symptom rebound group (P = 0.286) and between the persistent symptoms group and symptom rebound group (P = 0.068) were not statistically significant (Table 8).

**TABLE 8:** Comparison of prognostic outcomes among trajectory groups.

Prognostic outcome	Rapid recovery group (n = 82)	Slow recovery group (n = 62)	Persistent symptoms group (n = 48)	Symptom rebound group (n = 24)	Statistic	P Value	Post-hoc comparison
Primary outcomes							
Re-exacerbation occurrence [n(%)]‡	52(63.4)	51(82.3)	39(81.3)	20(83.3)	χ^2^ = 12.86	0.005	A < B,C,D
Median time to first re-exacerbation (days, 95%CI)	248(218–278)	156(124–188)	78(58–98)	102(72–132)	Log-rank χ^2^ = 38.62	<0.001	A > B,C,D; B > C
Re-exacerbations within 12 months (times, M[P25,P75])	1[0, 2]	2[1, 3]	3[2, 4]	2[1, 4]	H = 48.62	<0.001	A < B,C,D; B < C
Rehospitalizations within 12 months (times, M[P25,P75])	0[0, 1]	1[0, 2]	2[1, 3]	1[0, 2]	H = 38.94	<0.001	A < B,C,D; B < C
Secondary outcomes							
12-month all-cause mortality [n(%)]	2(2.4)	3(4.8)	8(16.7)	2(8.3)	—	0.008†	—§
Emergency department visits (times, M[P25,P75])	0[0, 1]	1[0, 2]	2[1, 3]	1[1, 2]	H = 36.28	<0.001	A < C,D; B < C
SGRQ total score (points, x̄±s)							
Baseline	48.6 ± 12.4	52.4 ± 13.2	58.6 ± 14.8	54.2 ± 13.6	F = 18.42	<0.001	A < C
12 months	38.2 ± 10.8	46.8 ± 12.4	56.2 ± 14.2	56.8 ± 14.6	F = 32.68	<0.001	A < B,C,D; B < C,D

SGRQ, St. George’s Respiratory Questionnaire; †Fisher’s exact test; ‡Re-exacerbation rate includes patients who experienced exacerbation before death, with denominators being total patient numbers in each group; §Small number of deaths in each group (2–8 cases) with limited statistical power; pairwise comparisons not performed; A = Rapid recovery group, B = Slow recovery group, C = Persistent symptoms group, D = Symptom rebound group; Post-hoc pairwise comparisons used Bonferroni correction (normally distributed continuous variables), Dunn-Bonferroni correction (non-normally distributed continuous variables), Log-rank test (survival data), or χ^2^ partitioning (categorical variables); corrected P < 0.0083 considered statistically significant.

#### 3.3.2 Secondary outcomes

For 12-month all-cause mortality, 15 patients (6.9%) died overall, including 2 (2.4%) in the rapid recovery group, 3 (4.8%) in the slow recovery group, 8 (16.7%) in the persistent symptoms group, and 2 (8.3%) in the symptom rebound group, with statistically significant differences among groups (Fisher’s exact test, P = 0.008). Given the small number of deaths in each group (2–8 cases), statistical power was limited; therefore, pairwise comparisons were not performed, and the above results are reported descriptively only.

For emergency department visits, the rapid recovery group had a median of 0 (IQR: 0–1), slow recovery group 1 (IQR: 0–2), persistent symptoms group 2 (IQR: 1–3), and symptom rebound group 1 (IQR: 1–2), with statistically significant differences among groups (H = 36.28, P < 0.001). Post-hoc pairwise comparisons (Dunn-Bonferroni correction) showed: the rapid recovery group had significantly fewer visits than the persistent symptoms group (P < 0.001) and symptom rebound group (P = 0.004); the slow recovery group had significantly fewer than the persistent symptoms group (P = 0.005); differences between the rapid recovery group and slow recovery group (P = 0.024), between the slow recovery group and symptom rebound group (P = 0.268), and between the persistent symptoms group and symptom rebound group (P = 0.124) were not statistically significant.

For SGRQ total scores, there were statistically significant differences among groups at baseline (F = 18.42, P < 0.001) and at 12-month follow-up (F = 32.68, P < 0.001). The rapid recovery group’s SGRQ total score decreased from 48.6 ± 12.4 at baseline to 38.2 ± 10.8 at 12 months; the slow recovery group decreased from 52.4 ± 13.2 to 46.8 ± 12.4; the persistent symptoms group decreased from 58.6 ± 14.8 to 56.2 ± 14.2; and the symptom rebound group increased from 54.2 ± 13.6 to 56.8 ± 14.6. At baseline, post-hoc pairwise comparisons (Bonferroni correction) showed: the rapid recovery group had significantly lower SGRQ total score than the persistent symptoms group (P < 0.001); differences between other group pairs did not reach statistical significance after correction (P > 0.0083). At 12 months, post-hoc pairwise comparisons showed: the rapid recovery group had significantly lower SGRQ total score than the slow recovery group (P = 0.002), persistent symptoms group (P < 0.001), and symptom rebound group (P < 0.001); the slow recovery group had significantly lower scores than the persistent symptoms group (P = 0.003) and symptom rebound group (P = 0.004); the difference between the persistent symptoms group and symptom rebound group was not statistically significant (P = 0.862) (Table 9).

**TABLE 9:** Supplementary summary of P-values for pairwise comparisons of prognostic outcomes.

Comparison Groups	Re-exacerbation Rate	Time to First Re-exacerbation (Log-rank)	Re-exacerbations within 12 Months	Rehospitalizations within 12 Months	ED Visits	SGRQ Baseline	SGRQ 12 Months
A vs. B	0.006	<0.001	0.003	0.004	0.024	0.086	0.002
A vs. C	0.008	<0.001	<0.001	<0.001	<0.001	<0.001	<0.001
A vs. D	0.006	<0.001	0.005	0.006	0.004	0.068	<0.001
B vs. C	0.524	0.002	0.006	0.003	0.005	0.042	0.003
B vs. D	0.686	0.048	0.324	0.286	0.268	0.486	0.004
C vs. D	0.586	0.186	0.142	0.068	0.124	0.286	0.862

A = Rapid recovery group, B = Slow recovery group, C = Persistent symptoms group, D = Symptom rebound group; ED, emergency department; Corrected P < 0.0083 considered statistically significant.

### 3.4 Association analysis between symptom trajectory types and prognosis

#### 3.4.1 Cox proportional hazards regression analysis

Schoenfeld residual tests showed that all variables met the proportional hazards assumption (all P > 0.05). Using the rapid recovery group as reference, univariable Cox regression analysis showed that the slow recovery group (Hazard Ratio [HR] = 1.86, 95%CI: 1.26–2.74, P = 0.002), persistent symptoms group (HR = 3.42, 95%CI: 2.28–5.13, P < 0.001), and symptom rebound group (HR = 2.68, 95%CI: 1.62–4.43, P < 0.001) all had elevated first re-exacerbation risk.

After adjusting for age, sex, FEV1%pred, number of exacerbations in the previous year, and CCI, multivariable Cox regression analysis showed that, compared with the rapid recovery group, the slow recovery group (adjusted HR [aHR] = 1.62, 95%CI: 1.08–2.42, P = 0.019), persistent symptoms group (aHR = 2.58, 95%CI: 1.68–3.96, P < 0.001), and symptom rebound group (aHR = 2.24, 95%CI: 1.32–3.80, P = 0.003) still had elevated first re-exacerbation risk (Table 10).

**TABLE 10:** Association analysis between symptom trajectory types and first Re-exacerbation risk.

Trajectory type	Univariable cox regression			Multivariable cox regression†			Fine-gray model‡		
​	HR	95%CI	P value	aHR	95%CI	P value	aSHR	95%CI	P value
Rapid recovery group	1	—	—	1	—	—	1	—	—
Slow recovery group	1.86	1.26–2.74	0.002	1.62	1.08–2.42	0.019	1.58	1.05–2.38	0.028
Persistent symptoms group	3.42	2.28–5.13	<0.001	2.58	1.68–3.96	<0.001	2.42	1.56–3.76	<0.001
Symptom rebound group	2.68	1.62–4.43	<0.001	2.24	1.32–3.80	0.003	2.18	1.28–3.72	0.004

HR, hazard ratio; aHR, adjusted hazard ratio; aSHR, adjusted subdistribution hazard ratio; CI, confidence interval; †Adjusted for age, sex, FEV1%pred, number of exacerbations in previous year, CCI; ‡Death as competing risk event, adjustment variables same as multivariable Cox regression.

#### 3.4.2 Competing risk model analysis

Considering death as a competing risk event, Fine-Gray competing risk model analysis results were consistent with Cox regression. Compared with the rapid recovery group, the slow recovery group (adjusted Subdistribution Hazard Ratio [aSHR] = 1.58, 95%CI: 1.05–2.38, P = 0.028), persistent symptoms group (aSHR = 2.42, 95%CI: 1.56–3.76, P < 0.001), and symptom rebound group (aSHR = 2.18, 95%CI: 1.28–3.72, P = 0.004) all had elevated subdistribution hazard of first re-exacerbation (Table 10).

#### 3.4.3 Negative binomial regression analysis

Using the rapid recovery group as reference, negative binomial regression analysis showed that, after adjusting for confounders, the slow recovery group (adjusted Incidence Rate Ratio [aIRR] = 1.54, 95%CI: 1.12–2.12, P = 0.008), persistent symptoms group (aIRR = 2.36, 95%CI: 1.68–3.32, P < 0.001), and symptom rebound group (aIRR = 1.82, 95%CI: 1.18–2.80, P = 0.007) all had increased re-exacerbation counts within 12 months.

For rehospitalizations within 12 months, compared with the rapid recovery group, the slow recovery group (aIRR = 1.68, 95%CI: 1.08–2.62, P = 0.021), persistent symptoms group (aIRR = 2.86, 95%CI: 1.82–4.50, P < 0.001), and symptom rebound group (aIRR = 1.92, 95%CI: 1.14–3.24, P = 0.014) all had increased rehospitalization counts (Table 11).

**TABLE 11:** Negative binomial regression analysis of symptom trajectory types and Re-exacerbation and rehospitalization counts within 12 Months.

Trajectory type	Re-exacerbations within 12 Months			Rehospitalizations within 12 Months		
​	aIRR	95%CI	P value	aIRR	95%CI	P value
Rapid recovery group	1	—	—	1	—	—
Slow recovery group	1.54	1.12–2.12	0.008	1.68	1.08–2.62	0.021
Persistent symptoms group	2.36	1.68–3.32	<0.001	2.86	1.82–4.50	<0.001
Symptom rebound group	1.82	1.18–2.80	0.007	1.92	1.14–3.24	0.014

aIRR, adjusted incidence rate ratio; CI, confidence interval; Adjusted for age, sex, FEV1%pred, number of exacerbations in previous year, CCI.

### 3.5 Independent predictors of symptom trajectory types

Using trajectory grouping as the dependent variable (rapid recovery group = 0, non-rapid recovery group = 1), multivariable logistic regression was performed to identify independent predictors of non-rapid recovery trajectory. Results showed that higher admission CAT score (Odds Ratio [OR] = 1.18, 95%CI: 1.08–1.29, P < 0.001), lower FEV1%pred (OR = 0.96, 95%CI: 0.93–0.99, P = 0.006), ≥2 exacerbations in the previous year (OR = 2.42, 95%CI: 1.36–4.31, P = 0.003), higher admission CRP (OR = 1.01, 95%CI: 1.003–1.018, P = 0.012), and current smoking (OR = 2.15, 95%CI: 1.12–4.13, P = 0.021) were independent predictors of non-rapid recovery trajectory (Table 12).

**TABLE 12:** Multivariable logistic regression analysis of non-rapid recovery trajectory.

Variable	β	SE	Wald χ^2^	OR	95%CI	P Value
Admission CAT score (per 1-point increase)	0.166	0.046	13.02	1.18	1.08–1.29	<0.001
FEV1%pred (per 1% increase)	−0.041	0.015	7.48	0.96	0.93–0.99	0.006
≥2 exacerbations in previous year	0.884	0.295	8.98	2.42	1.36–4.31	0.003
Admission CRP (per 1 mg/L increase)	0.01	0.004	6.25	1.01	1.003–1.018	0.012
Current smoking (vs. non-current smoker)	0.765	0.332	5.31	2.15	1.12–4.13	0.021
Age (per 1-year increase)	0.024	0.018	1.78	1.02	0.99–1.06	0.182
Male (vs. female)	0.286	0.362	0.62	1.33	0.65–2.71	0.43
CCI score (per 1-point increase)	0.142	0.098	2.1	1.15	0.95–1.40	0.147

CAT, COPD, assessment test; FEV1%pred, FEV1 percent predicted; CRP, C-reactive protein; CCI, charlson comorbidity index; SE, standard error; OR, odds ratio.

## 4 Discussion

This study used GBTM to identify 4 distinct symptom trajectory types during AECOPD recovery, with each trajectory type closely associated with prognosis. This finding provides a new perspective for understanding heterogeneity in AECOPD recovery (Machado et al., 2023). Notably, the observed inter-individual differences in recovery may be partly attributable to variation in the degree of systemic inflammatory activation at the time of acute exacerbation: patients with more pronounced inflammatory responses—reflected by higher admission CRP, PCT, and respiratory SOFA sub-score (derived from PaO2/FiO2) — tended to cluster into slower or non-recovery trajectories. These patterns are consistent with the Sepsis-3 framework, wherein dysregulated host inflammatory responses to infection drive organ dysfunction and may prolong recovery. It should be noted that complete SOFA-based Sepsis-3 classification was not feasible due to missing sub-score data; this is a limitation of the current study and CRP/PCT are used as surrogate indicators of systemic inflammatory burden.

Previous studies have largely viewed AECOPD recovery as a single process, overlooking inter-individual differences (Xu et al., 2024). This study found that approximately 38% of patients exhibited a rapid recovery pattern, while over 60% had suboptimal recovery, similar to the proportion of delayed recovery reported in international cohort studies (Halpin et al., 2023). Patients in the persistent symptoms and symptom rebound groups had significantly elevated re-exacerbation risk, suggesting that recovery trajectories can serve as an important basis for prognostic stratification (Ji et al., 2023).

This study found that admission CAT score, FEV1%pred, history of frequent exacerbations, CRP level, and smoking status were independent predictors of non-rapid recovery trajectory. The association between baseline symptom severity and recovery trajectory has been confirmed in multiple studies (Ellingsen et al., 2024; Ding et al., 2025). The degree of pulmonary function impairment reflects disease severity and is negatively correlated with recovery capacity (Backman et al., 2024); notably, the FEV1%pred gradient across the four trajectory groups (52.6%, 44.2%, 36.8%, and 40.5% for rapid recovery, slow recovery, persistent symptoms, and symptom rebound groups, respectively) provides objective physiological support that these phenotypes reflect genuine differences in underlying disease severity. A history of frequent exacerbations suggests persistent airway inflammation, which may affect the symptom recovery process (Kortekaas et al., 2024). Elevated admission CRP has been associated with poor COPD prognosis (Zhu et al., 2025). Similarly, PCT was significantly elevated in the persistent symptoms group compared to the rapid recovery group; in the Sepsis-3 framework, PCT elevation reflects bacterial infection-driven dysregulation of the host inflammatory response consistent with sepsis-associated immune activation, which may impair airway mucosal repair and prolong symptom resolution. Importantly, CRP and PCT were measured only at admission; patients with initially elevated but rapidly normalizing markers may have fundamentally different recovery biology from those with sustained elevation. Serial measurements aligned with the Sepsis-3 monitoring paradigm—including SOFA reassessment at discharge and early post-discharge timepoints—would be necessary to fully characterize inflammatory dynamics and their relationship to trajectory type. Current smoking delays symptom improvement by exacerbating airway inflammation and impairing mucosal repair (Lin and Li, 2023).

The symptom rebound group exhibited a unique “improvement-then-worsening” pattern, consistent with the “pseudo-recovery” phenomenon described in some studies (Goto et al., 2018). The short-term symptom improvement in this group after discharge may mask underlying problems, leading to insufficient follow-up or reduced treatment adherence (Case and Eakin, 2023). In this cohort, symptom re-worsening in the rebound group occurred predominantly between weeks 4 and 8 post-discharge, with respiratory tract re-infection and early discontinuation of inhaled therapy being the most commonly documented precipitants; the small sample size (n = 24) precludes formal analysis of these associations. The inflammatory marker profile of the rebound group (PCT 0.24 ng/mL; CRP 54.6 mg/L) was intermediate rather than distinctly elevated relative to other non-rapid recovery groups, which limits the proposed immune dysregulation hypothesis for this subgroup. The wide confidence intervals for the rebound group in Cox regression (aHR 2.24, 95%CI: 1.32–3.80) reflect this limited sample, and conclusions for this phenotype should be regarded as exploratory. Nonetheless, dynamic symptom monitoring within 4 weeks post-discharge is important, particularly for patients showing initial improvement (Li et al., 2023).

From a pathophysiological perspective, and consistent with the Sepsis-3 framework, AECOPD-precipitating infections can drive systemic inflammatory cascades that fulfill the criteria of life-threatening organ dysfunction caused by a dysregulated host response to infection (acute SOFA score increase ≥2). This inflammatory milieu amplifies local airway injury and contributes to systemic consequences including respiratory muscle fatigue and cardiovascular strain, all of which may prolong recovery. The elevated PCT and CRP levels observed in the persistent symptoms group in this study are consistent with sepsis-associated immune dysregulation as a contributor to suboptimal recovery trajectories. Although complete SOFA-based Sepsis-3 classification was not performed in this study—hepatic, renal, cardiovascular, coagulation, and neurological sub-scores were not collected—the available respiratory SOFA data (PaO2/FiO2) and inflammatory biomarkers provide partial support for this mechanistic hypothesis. Clinically, incorporating full SOFA assessment at admission alongside CRP and PCT measurement would enable formal Sepsis-3 classification and more precise identification of patients at elevated risk for non-rapid recovery. Future prospective studies should collect complete SOFA sub-scores to enable this classification. Additionally, Cox and Fine-Gray models in this study adjusted only for baseline variables; post-discharge management intensity was not captured as a time-varying covariate, and patients recognized as higher-risk may have received more intensive post-discharge care, potentially attenuating the observed prognostic differences—a recognized confounding limitation of observational cohort studies.

This study has several limitations. First, the single-center design from a TCM hospital setting may limit generalizability; TCM adjunctive therapies were not systematically tracked and their potential influence on trajectory cannot be excluded. Second, exclusion of patients requiring invasive ventilation or dying within 48 h may have introduced survivorship bias, underrepresenting the most severely ill. Third, the symptom rebound subgroup (n = 24) is underpowered, yielding wide confidence intervals; these results are exploratory. Fourth, no *a priori* power calculation was performed, and the event-per-variable ratio for some subgroup analyses falls below recommended thresholds. Fifth, GBTM relied on patient-reported CAT and mMRC scores without serial objective physiological measures; the FEV1%pred gradient provides partial objective support but serial measures (e.g., 6-min walk distance) are needed. Sixth, although Sepsis-3 criteria (Singer et al., 2016) define sepsis as an acute SOFA score increase ≥2 points due to a dysregulated host response to infection, complete SOFA-based classification was not feasible in this study: only the respiratory sub-score (PaO2/FiO2) was available; hepatic (bilirubin), renal (creatinine), cardiovascular (MAP/vasopressors), coagulation (platelets), and neurological (GCS) sub-scores were not collected. CRP and PCT were therefore used as surrogate biomarkers of sepsis-related inflammatory burden alongside partial SOFA data. Future prospective studies must collect complete SOFA data at admission, 24 h, and discharge to enable rigorous Sepsis-3 classification and its integration with trajectory modeling. Seventh, inflammatory biomarkers were measured only at admission; serial measurements would better characterize inflammatory dynamics. Eighth, antibiotic and corticosteroid use were recorded as binary variables, and sputum culture data were incomplete (148/216, 68.5%). Ninth, post-discharge management intensity was not captured as a time-varying covariate in survival models (Kim et al., 2024; Heros et al., 2023). Future multicenter prospective studies incorporating serial objective monitoring, complete SOFA-based Sepsis-3 classification, full microbiological data, and time-varying treatment covariates are needed to address these limitations (Gisbert and Chaparro, 2024; Hassan et al., 2025).

In conclusion, symptom trajectories during AECOPD recovery demonstrate clear heterogeneity, and elevated admission systemic inflammatory burden—reflected by CRP, PCT, and available respiratory SOFA data—is associated with non-rapid recovery trajectories consistent with sepsis-related immune dysregulation as described by Sepsis-3 criteria. Non-rapid recovery is associated with shorter time to re-exacerbation, higher rehospitalization frequency, and worse quality of life at 12 months. As complete SOFA-based Sepsis-3 classification was not feasible in this study, CRP and PCT were used as surrogate biomarkers of systemic inflammatory burden; future studies should integrate formal Sepsis-3 assessment. Trajectory-based prognostic stratification combined with Sepsis-3-informed inflammatory burden assessment may facilitate development of individualized follow-up management strategies.

## Data Availability

The datasets generated and analyzed during the current study are not publicly available due to privacy and ethical restrictions, but are available from the corresponding author on reasonable request.
